# Exploration of the Optimal Minimum Lymph Node Count after Colon Cancer Resection for Patients Aged 80 Years and Older

**DOI:** 10.1038/srep38901

**Published:** 2016-12-12

**Authors:** Xu Guan, Wei Chen, Zheng Jiang, Zheng Liu, Dazhuang Miao, Hanqing Hu, Zhixun Zhao, Runkun Yang, Xishan Wang

**Affiliations:** 1 Department of Colorectal Surgery, The Second Affiliated Hospital of Harbin Medical University, Harbin, China; 2 Department of Colorectal Surgery, Cancer Institute & Hospital, Chinese Academy of Medical Sciences, Peking Union Medical College, Beijing, China; 3 Follow up center, The Second Affiliated Hospital of Harbin Medical University, Harbin, China; 4 Department of Colorectal Surgery, The Affiliated Tumor Hospital of Harbin Medical University, Harbin, China

## Abstract

The elderly colon cancer (CC) patients are increasing and represent a heterogeneous patient group. The objectives of this study were to identify the features of lymph node examination and to explore the optimal minimum lymph node count after CC resection for patients aged ≥80. Using the Surveillance, Epidemiology, and End Results (SEER) database, we identified 65719 CC patients in stage I-III between 2004 and 2012, 26.0% of patients were aged ≥80. The median node count decreased with increasing age, which were 25.5, 20.2, 17.8 and 16.9 for patients aged 20–39, 40–59, 60–79, and ≥80. The rate of ≥12 nodes and the rate of node positivity for patients aged ≥80 were obviously lower than younger patients. Using X-tile analysis, we determined 9 nodes as the optimal node count for patients aged ≥80. Then, we compared the 5-year cancer specific survival (CSS) between patients with ≥9 nodes and <9 nodes. The results showed the 5-year CSSs were improved for patients with ≥9 nodes. Furthermore, the rate of node positivity and survival under the 9-node measure were equal to 12-node measure. Therefore, the lymph node examination should be discriminately evaluated for elder patients, and 9-node measure was available for patients aged ≥80.

Elderly patients currently account for a significant proportion of patients who are diagnosed with colon cancer (CC), which bring a challenge of dealing with an aging population to medical oncologists^1^. the US Preventive Services Task Force guidelines have showed that the percentage of colorectal cancer patients aged ≥75 increased from 29% to 40% between 1973 and 2007^2^. With the increasing and large proportion of the elderly CC patients, more attention should be paid for this group of patients. However, many questions regarding to the elder patients, especially for patients aged ≥80, were still not well defined.

Accurate cancer staging is highly dependent on sufficient number of lymph nodes examination to detect the positive nodes^3^. Positive nodes are closely associated with adverse prognosis and more appropriate use of adjuvant chemotherapy for CC patients^4^,^5^,^6^. Therefore, numerous studies have attempted to explore the optimal minimum lymph node count that contribute to improved tumor staging and survival outcomes, but individual studies varied widely in their recommendations for lymph node count to accurately detect the positive nodes^7^,^8^,^9^. The National Comprehensive Cancer Network (NCCN) guideline and the American Society of Clinical Oncology (ASCO) advocate that 12 regional lymph nodes should be the necessary minimum node count for quality evaluation of CC resection. However, lymph node examination of CC was influenced by several factors, including patient-, tumor-, and treatment-related factors. Among them, age has been considered as one important influential factor on node examination^10^,^11^,^12^. The disparity in nodal examination gradually begs the question of whether the 12-node measure is an appropriate threshold for all CC patients.

Elder CC patients have distinct characteristics that need to be taken into account regarding lymph node evaluation, especially for patients aged ≥80^13^,^14^. However, little attention has been paid to this issue, which contributed to a distinct lack of data on these elder patients. Accordingly, with data from the Surveillance, Epidemiology, and End Results (SEER) database, we firstly assessed the impact of age on lymph nodal evaluation of CC resection. Secondly, we attempted to explore the minimum optimal node count for patients aged ≥80, instead of the standard 12-node measure. Finally, we evaluated the availability of this revised node measure by comparing the node positivity rate and long-term survival with the 12-node measure.

## Results

### Patient characteristics

We totally identified 65719 patients diagnosed with stage I-III CC from 2004 to 2012 in the SEER database, 17058 patients (26.0%) were aged 80 years and older. Baseline characteristics significantly differed among four subgroups (Table 1). Patients aged ≥80 were more common seen in female patients (64.1%), this proportion was obviously higher than other three subgroups. The proportion of stage III patients gradually decreased with age, varying from 51.1% in aged 20–39 subgroup to 32.6% in aged ≥80 subgroup. Overall, the largest proportions were white (80.4%), adenocarcinoma (86.5%), grade II (67.0%) right side (82.0%) and T3/T4 (71.2%), distributions of these characteristics were fairly uniform across four subgroups.

### The comparisons of lymph node evaluation among different age subgroups

**Figure 1:**
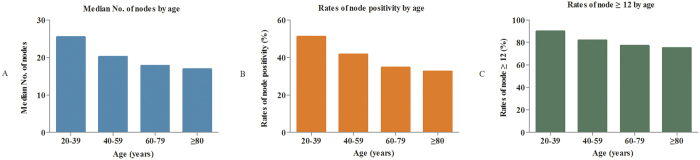
(**A**) Comparisons of median number of lymph nodes among different age subgroups. (**B**) Comparisons of node positivity rate among different age subgroups. (**C**) Comparisons of rate of ≥12 nodes among different age subgroups.

In this study, patients with adequate lymph node count (≥12) accounted for 77.9% in all patients, and hence it was confidently staged according to their nodal status. The median number of lymph node count was decreased with increasing age, which were 25.5, 20.2, 17.8 and 16.9 for patients aged 20–39, 40–59, 60–79, and ≥80, respectively (P < 0.001) (Fig. 1A). For lymph node positivity, the proportion of patients with at least one node positive were 51.1%, 41.6%, 34.6%, and 32.6% for patients aged 20–39, 40–59, 60–79, and ≥80 (P < 0.001) (Fig. 1B). The rate of ≥12 nodes was the lowest for patients aged ≥80 compared with the younger patients (P < 0.001) (Fig. 1C). These results showed that patients aged ≥80 were associated with poor lymph node harvest and lower rate of node positivity. Therefore, the elderly patient certainly faced greater challenge of harvesting adequate lymph nodes (≥12 nodes) compared with younger patients. In other words, the standard 12-node measure may be not reasonable to be equally required for the elderly patients as young patients. Accordingly, we tentatively explored an optimal cut-off node count for patients aged ≥80, instead of 12 nodes.

### Identification of the optimal cut-off point of lymph node count for patients aged ≥80

**Figure 2:**
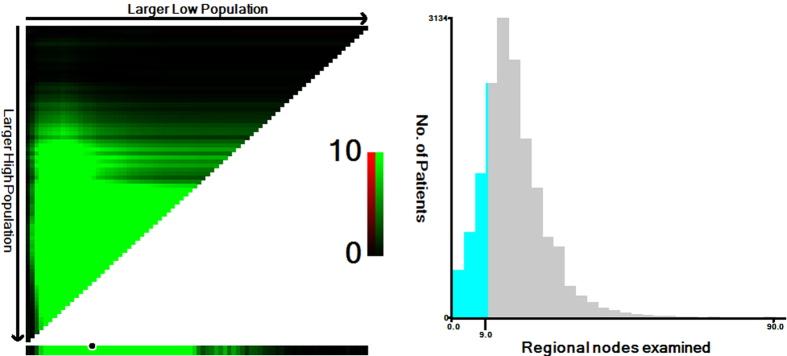
(**A**) X-tile plots for number of lymph nodes constructed by patients aged ≥80. The plots show the χ^2^ log-rank values produced, dividing them into 2 groups by the cutoff point 9. The brightest pixel represents the maximum χ^2^ log-rank value. The data are represented graphically in a right-triangular grid where each point represents the data from a given set of divisions. The vertical axis represents all possible “high” populations, with the size of the high population increasing from top to bottom. The horizontal axis represents all possible “low” populations, with the size of the low population increasing from left to right. Data along the hypotenuse represent results from a single cut-point that divides the data into high or low subsets. (**B**) The distribution of number of patients aged ≥80 according to lymph nodes count. Number of lymph nodes ranged from 0 to 90.

We applied X-tile analysis to determine the optimal cut-off node count for prediction of CSS according to different lymph node count. The result showed that 9 was the optimal cut-off node count for patients aged ≥80 (P < 0.001) (Fig. 2). Therefore, this cut-off value was used as prognostic factor for further analysis in patients aged ≥80.

### Prognostic impact of the 9-node measure on CCS for patients aged ≥80

**Figure 3:**
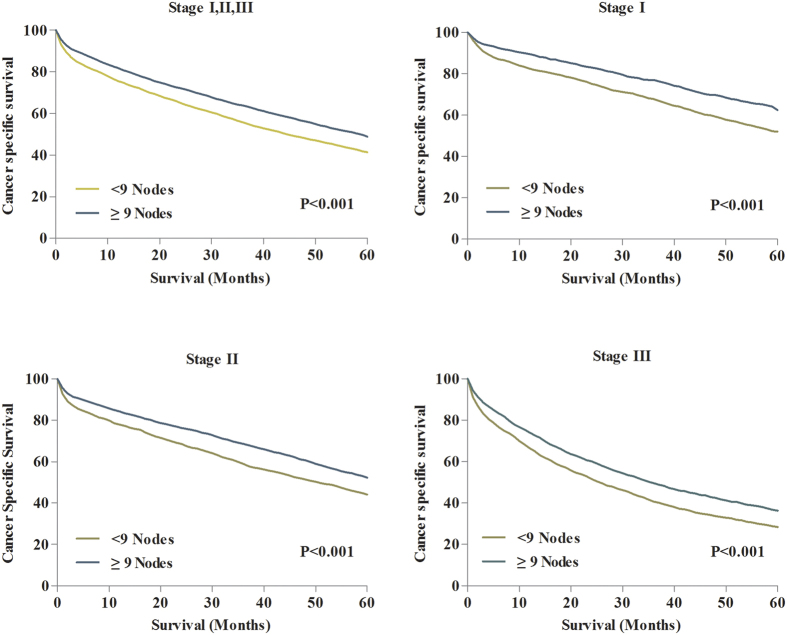
(**A**) 5-year CSSs in all patients between lymph node count ≥9 and <9. (**B**) 5-year CSSs in patients with stage I between lymph node count ≥9 and <9. (**C**) 5-year CSSs in patients with stage II between lymph node count ≥9 and <9. (**D**) 5-year CSSs in patients with stage III between lymph node count ≥9 and <9.

The median CSSs were 55.0 months for patients with ≥9 nodes, and 39.0 months for patients with <9 nodes. The 5-year CSS was 46.7% for patients with ≥9 nodes and 38.6% for those with <9 nodes (P < 0.001) (Fig. 3A). Then, we separately evaluated the effect of 9-node measure on CSS of patients in different tumor staging. The results indicated that patients in stage I, II and III all obtained survival benefit from ≥9 nodes compared with patients who examined <9 nodes (Fig. 3B–D).

### Identification of risk factors for survival for patients aged ≥80

Using univariate and multivariate regression analyses, we identified the risk factors that associated with long-term survival outcomes for patients aged ≥80. The results showed that retrieval of <9 nodes was identified as independent adverse prognostic factors in patients aged ≥80 (Table 2). In addition, characteristics including black, male, stage II/III, T3/4 stage, N1/2 and grade II/III/IV were all identified independent risk factors for survival in patients aged ≥80.

### Comparisons of the rate of node positivity and survival between 9-node measure and 12-node measure

**Figure 4:**
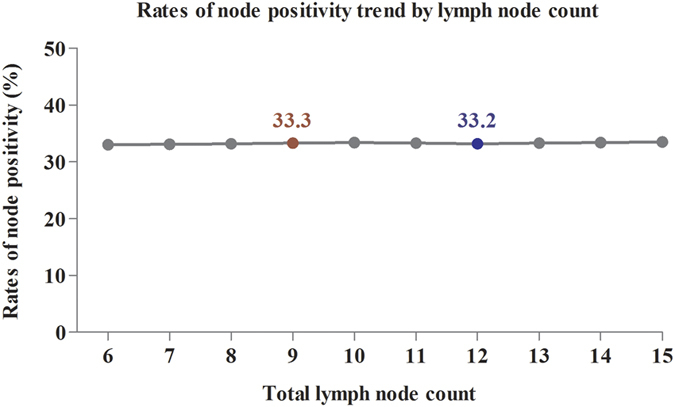
The trend of node positivity rate according to lymph node count from **≥**6 to **≥**15.

Adequate lymph node retrieval was higher related to accurate tumor staging and improved long-term survival. To confirm the value of 9-node measure for patient aged ≥80, we separately evaluate the effect of lymph node count on the rate of node positivity and long-term survival. Firstly, we separately calculated the rate of node positivity based on different node count from ≥6 to ≥15. The result showed that the rate of node positivity changed between 33.0% and 33.5% according to the node count from ≥6 to ≥15 (Fig. 4). The rate of node positivity was 33.3% for patient with ≥9 nodes, which was higher than patients with ≥12 nodes (33.2%). The detailed information was shown in Supplementary Table 1. This result indicated that the 9-node measure did not reduce the rate of node positivity compared with the standard 12-node measure for patients aged ≥80. Due to patients with positive lymph node are belong to stage III, the rate of node positivity is equal to the proportion of stage III patients in all patients. For the elderly patients, the proportion of stage III patients was not reduced by using the 9-node measure. Examining ≥9 nodes is therefore enough to determine tumor stage and could be considered as adequate surgery.

**Figure 5:**
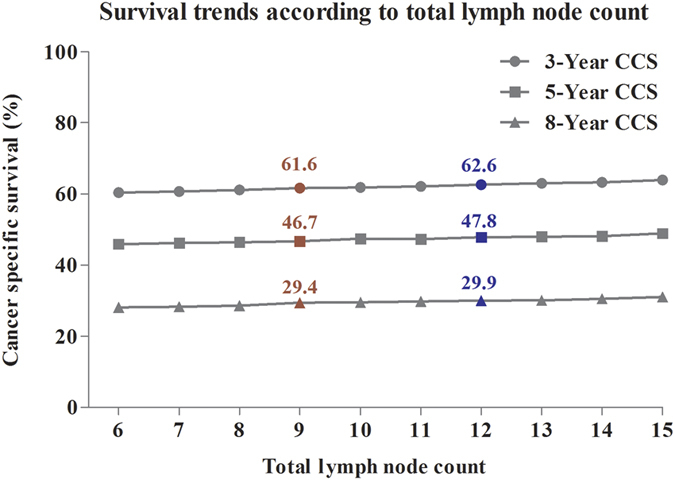
The trend of long-term survivals according to lymph node count from **≥**6 to **≥**15.

In addition, we assessed the effect of node count on the long-term survival outcomes. The 3-, 5- and 8-year CCSs were separately calculated according to node count from ≥6 to ≥15. For patients with ≥9 nodes, the 3-, 5- and 8-year CSSs were 61.6%, 46.7% and 29.4%. For patients with ≥12 nodes, the 3-, 5- and 8-year CSSs were 62.6%, 47.8% and 29.9% (Fig. 5). Therefore, the results showed that the long-term survivals were similar between these two groups. This result suggested that although the increased node count was associated with better survival outcomes, the long-term survival of patients with ≥9 nodes was not obviously decreased compared with those who examined ≥12 nodes for patients aged ≥80.

### Survival comparison in patients with different nodes examined

Furthermore, we performed the survival comparison in patients with different nodes examined (<9 nodes vs. 9–12 nodes vs. ≥12 nodes). The Supplementary Figure 1 showed that the 5-year CSS of patients with ≥12 nodes examined was 47.8%, which was significantly higher than patients with 9–12 nodes (40.5%) and patients with <9 nodes (38.6%). This result suggested that the number of nodes examined was positively associated with survival outcomes for the elderly patients.

## Discussion

CC patients aged ≥80 represented a large proportion in all patients. Here, we totally identified 17058 CC patients aged ≥80, which accounted for 26.0% in all patients from 2004 to 2012. Elder patients, especially for patients aged ≥80, not only has been thought as an independent prognostic factor regarding with survival, but also has been associated with different clinical and histological features^9^,^13^,^14^,^15^. Therefore, patients aged ≥80 were highly heterogeneous population, which should be discriminately evaluated in the consideration of cancer diagnosis and treatment.

Lymph node examination after CC resection is considered to be one crucial factor in assessing the accuracy of tumor staging, which determine further therapeutic planning and prognosis. Patients with positive nodes have to accept adjuvant chemotherapy regarding with the higher risk of tumor recurrence and metastasis^16^. Current guidelines advocate that the lymph node examination should meet the requirement of at least 12 nodes examined in CC specimen. However, the lymph node evaluation was highly heterogeneous, which was affected by several factors. Age was thought to be an important factor that influenced the lymph node examination of CC. The CC patients who aged ≥80, had obviously lower lymph node harvest compared with the younger patients^11^. In this study, the results showed that the median number of lymph nodes examined was 16.9 for patients aged ≥80, which was significantly fewer than other three younger patient groups. A possible explanation for this result may be related to a weaker immunological response to a malignant tumor in the elderly^17^. In addition, older age might also contribute to more limited resections compared with younger patients^18^,^19^. Whatever the potential reasons for poor node harvest in the elderly, it was the fact that a retrieval of ≥12 nodes was certainly difficult for this group of patients. Therefore, it might be unreasonable to request that the 12-node measure was equally used for both patients aged ≥80 and the younger patients.

In this study, we tentatively explore the optimal cut-off node count, instead of 12 nodes, for patients aged ≥80 based on the prediction of CSS. Our results identified that 9 nodes was the optimal cut-off point for patients aged ≥80. All CC patients in stage I, II and III all could obtain much survival benefit from examining ≥9 nodes, and examining <9 nodes was also identified as independent adverse prognostic factors in patients aged ≥80. In addition, 9-node measure did not decrease the chance of obtaining positive lymph node compared with the 12-node measure. Hence, the use of the 9-node measure might be more reasonable for patients aged ≥80.

In identifying the difference of lymph node examination for CC patients aged ≥80, the SEER databases provide sufficient CC cases with uniformly collected data, which highly represented the CC patients across the United States. However, there had some potential limitations in SEER database including lack of information regarding the adjuvant chemotherapy for CC patients, lack of central histological review, and lack of information on operative morbidity and comorbidities. The study may also be confounded by other contributing factors, such as general ASA status, which may indirectly affect the lymph node examination in the elderly patients. These noted limitations in SEER are common to most of other large epidemiological databases, which have been well addressed in literatures. Furthermore, the selection biases could not be avoided in this retrospective cohort study, because the elderly patients who underwent CC resection should be fitter, and the radical surgery of CC might be not regularly performed for this group of patients as young patients. Despite these limitations, SEER remains a valuable resource to analyze trends and patterns in patient characteristics, tumor features, cancer treatments, and survival outcomes.

In conclusion, this population-based study demonstrated that patients aged ≥80 accounted for a large proportion of CC patients, they had obviously lower rate of ≥12 nodes examined compared with the younger patients. Instead of 12-node measure, we identified that the 9-node measure may be more feasible and reasonable in patients aged ≥80. However, whether this finding could impact the adjuvant treatment decision-making for the elderly patient who cannot tolerate chemotherapy, more investigations of outcomes for this group of CC patients are still need to be performed.

## Materials and Methods

### Data sources

We extracted cancer data from the Surveillance, Epidemiology, and End Results (SEER) database between 2004 and 2012. The SEER database includes the information regarding cancer incidence, treatment and survival outcomes from 17 population-based cancer registries, which represented 28% of the US population^20^. Data collected from the SEER database do not require informed patient consent, because they were anonymized and de-identified prior to release.

This study was approved by the Ethics Committee of The Second Affiliated Hospital of Harbin Medical University institutional review board. The SEER database is openly accessed, and we have got permission to access the cancer data from the SEER database by National Cancer Institute, and the reference number was 11228-Nov2014. All methods were performed in accordance with the relevant guidelines and regulations of SEER database.

### Study population

We collected cases based on the International Classification of Diseases Oncology, Third Edition (ICD-O-3) codes for anatomic site (colon excluding rectum). We used the term “age” which was referred to “age at diagnosis” in SEER database. Patients were divided into four age subgroups by 20-year intervals, including 20–39, 40–59, 60–79, ≥80. Patients included in this study should undergo radical resection of the CC as the first course of therapy, which were more available and accurate for the lymph node evaluation. Race/ethnicity was categorized as white, black and Asian/Pacific Islander (API), including American Indian/Alaska Natives. Right CC included tumors being located at cecum, ascending colon, hepatic flexure and transverse colon. Left CC included tumors being located at splenic flexure, descending colon and sigmoid colon. The exclusion criteria should include patients: dead due to other causes, with an unknown number of nodes examined, aged <20 years, who received preoperative radiotherapy in the consideration of the decreased number of node examined, and who underwent a local procedure, partial colon resection or total colectomies.

### Statistical analysis

The comparisons of lymph node evaluation among different age subgroups were performed in three ways including the median number of lymph node, the rate of ≥12 lymph nodes and the rate of node positivity. All categorical variables were compared between groups using χ^2^ test. The cancer specific survival (CSS) was defined as the time from the CC diagnosis until cancer recurrence or metastasis, cancer-associated death and the end of follow up. The 5-year CSS was estimated with Kaplan-Meier method and log-rank test was used to compare the difference of CSS curves. Univariate and multivariate Cox’s regression model were performed to estimate hazard rate (HR) and exact 95% confidence intervals (CIs). P < 0.05 (two sides) was considered to be statistical significance. The statistical analyses were conducted with SPSS statistical software, version 20 (IBM Corp, Armonk, NY, USA).

X-tile plots is a new bio-informatics tool for biomarker assessment and outcome-based cut-point optimization, which illustrates the presence of substantial patients subpopulations and shows the robustness of the relationship between a marker and survival outcome by construction of a two dimensional projection of every possible subpopulation. X-tile plots divided population into different divisions based on every possible cutoff point. X-tile data are presented in a right triangular grid where each point represents a different cutoff point. The X-tile plots statistically test each divisions based on each cutoff point. All possible divisions of the cutoff point are assessed. Then, a χ^2^ value is calculated for every possible division of the population. The optimal cutoff node count for survival was calculated by selecting minimum P value with the maximum χ^2^ value.

## Additional Information

**How to cite this article**: Guan, X. *et al*. Exploration of the Optimal Minimum Lymph Node Count after Colon Cancer Resection for Patients Aged 80 Years and Older. *Sci. Rep.* **6**, 38901; doi: 10.1038/srep38901 (2016).

**Publisher's note:** Springer Nature remains neutral with regard to jurisdictional claims in published maps and institutional affiliations.

## Supplementary Material

Supplementary Information

## Figures and Tables

**Table 1:** Characteristics of CC patients in the SEER database: 2004–2012.

Characteristics	20–39 years N = 1271 (%)	40–59 years N = 13743 (%)	60–79 years N = 33647 (%)	≥80 years N = 17058 (%)	Total N = 65719 (%)
Gender					
Male	666 (52.4)	7444 (54.2)	16498 (49.0)	6119 (35.9)	30727 (46.8)
Female	605 (47.6)	6299 (45.8)	17149 (51.0)	10939 (64.1)	34992 (53.2)
Race					
White	922 (72.5)	9907 (72.1)	26996 (80.2)	14987 (87.9)	52812 (80.4)
Black	193 (15.2)	2652 (19.3)	4198 (12.5)	1111 (6.5)	8154 (12.4)
Others	142 (11.2)	1100 (8.0)	2341 (7.0)	930 (5.4)	4513 (6.9)
Unknown	14 (1.1)	84 (0.6)	112 (0.3)	30 (0.2)	240 (0.3)
AJCC Stage					
Stage I	148 (11.7)	3153 (22.9)	9038 (26.9)	3961 (23.2)	16300 (24.8)
Stage II	473 (37.2)	4879 (35.5)	12971 (38.5)	7535 (44.2)	25858 (39.3)
Stage III	650 (51.1)	5711 (41.6)	11638 (34.6)	5562 (32.6)	23561 (35.9)
Grade					
Grade I	70 (5.5)	1281 (9.3)	3114 (9.3)	1373 (8.0)	5838 (8.9)
Grade II	803 (63.2)	9449 (68.8)	22819 (67.8)	10997 (64.5)	44068 (67.0)
Grade III	315 (24.8)	2280 (16.6)	5968 (17.7)	3846 (22.6)	12409 (18.9)
Grade IV	45 (3.5)	267 (1.9)	697 (2.1)	445 (2.6)	1454 (2.2)
Unknown	38 (3.0)	466 (3.4)	1049 (3.1)	397 (2.3)	1950 (3.0)
Histology type					
Adenocarcinoma	1027 (80.8)	12011 (87.4)	29304 (87.1)	14521 (85.1)	56863 (86.5)
Mucous/signet-ring cell	230 (18.1)	1660 (12.1)	4112 (12.2)	2368 (13.9)	8370 (12.8)
Other types	14 (1.1)	72 (0.5)	231 (0.7)	169 (1.0)	486 (0.7)
Tumor location					
Left-sided colon	404 (31.8)	3597 (26.2)	5848 (17.4)	1973 (11.6)	11822 (18.0)
Right-sided colon	867 (68.2)	10146 (73.8)	27799 (82.6)	15085 (88.4)	53897 (82.0)
T stage					
T1/T2	199 (15.7)	3813 (27.7)	10459 (31.1)	4484 (26.3)	18955 (28.8)
T3/T4	1072 (84.3)	9930 (72.3)	23188 (68.9)	12574 (73.7)	46764 (71.2)
N stage					
N0	621 (48.9)	8032 (58.4)	22009 (65.4)	11496 (67.4)	42158 (64.1)
N1/N2	650 (51.1)	5711 (41.6)	11638 (34.6)	5562 (32.6)	23561 (35.9)

Others: American Indian/AK Native, Asian/Pacific Islander.

**Table 2:** Univariate and multivariate analyses for CC patients aged ≥80.

Characteristic		Univariate analysis		Multivariate analysis	
		HR [95% CI]	P	HR [95% CI]	P
Node examined	<9	1	<0.001	1	<0.001
	≥9	0.797 [0.764–0.832]		0.766 [0.733–0.800]	
Gender	Female	1	<0.001	1	<0.001
	Male	1.150 [1.101–1.202]		1.173 [1.122–1.226]	
Race	White	1	<0.001	1	<0.001
	Black	1.140 [1.048–1.240]		1.145 [1.052–1.246]	
	Others	0.749 [0.675–0.832]		0.726 [0.654–0.806]	
AJCC stage	Stage I	1	<0.001	1	<0.001
	Stage II	1.250 [1.178–1.327]		1.124 [1.016–1.249]	
	Stage III	1.983 [1.868–2.106]		1.353 [1.185–1.545]	
T stage	T1/T2	1	<0.001	1	<0.001
	T3/T4	1.480 [1.405–1.558]		1.532 [1.348–1.742]	
N stage	N0	1	<0.001	1	<0.001
	N1/N2	1.710 [1.636–1.787]		1.743 [1.678–1.797]	
Tumor location	Right-sided colon	1	0.123		
	Left-sided colon	1.053 [0.986–1.124]			
Histological type	Adenocarcinoma	1	0.143		
	Mucous/signet-ring cell	1.059 [0.997–1.125]			
Grade	Grade I	1	<0.001	1	<0.001
	Grade II	1.067 [0.983–1.158]		1.003 [0.924–1.090]	
	Grade III	1.336 [1.223–1.460]		1.159 [1.059–1.269]	
	Grade IV	1.662 [1.432–1.930]		1.442 [1.238–1.680]	

Others: American Indian/AK Native, Asian/Pacific Islander.
