# Supplementary material for: Exploration of the Optimal Minimum Lymph Node Count after Colon Cancer Resection for Patients Aged 80 Years and Older
Source: Sci Rep. 2016 Dec 12;6:38901. doi: 10.1038/srep38901 (PMC5150780; doi:10.1038/srep38901)
Supplement: Supplementary Information [file srep38901-s1.docx]

**Exploration of the Optimal Minimum Lymph Node Count after Colon Cancer Resection for Patients Aged 80 Years and Older**

Xu Guan^1,2^, Wei Chen^3^, Zheng Jiang^2^, Zheng Liu^2^, Dazhuang Miao^4^, Hanqing Hu^1^, Zhixun Zhao^1^, Runkun Yang^1^, Xishan Wang^1,2^

1 Department of Colorectal Surgery, The Second Affiliated Hospital of Harbin Medical University, Harbin, China.

2 Department of Colorectal Surgery, Cancer Institute & Hospital, Chinese Academy of Medical Sciences, Peking Union Medical College, Beijing, China.

3 Follow up center, The Second Affiliated Hospital of Harbin Medical University, Harbin, China.

4 Department of Colorectal Surgery, The Affiliated Tumor Hospital of Harbin Medical University, Harbin, China.

**Supplementary Table 1** The change of node positivity rate according to lymph node count.

| **Total lymph node count** | **No. of patients** | **No. of patients with positive nodes** | **Rate of node positivity** |
| --- | --- | --- | --- |
| **≥6** | 16022 | 5283 | 33.0% |
| **≥7** | 15662 | 5177 | 33.1% |
| **≥8** | 15223 | 5056 | 33.2% |
| **≥9** | 14715 | 4899 | 33.3% |
| **≥10** | 14156 | 4724 | 33.4% |
| **≥11** | 13490 | 4498 | 33.3% |
| **≥12** | 12792 | 4252 | 33.2% |
| **≥13** | 11707 | 3895 | 33.3% |
| **≥14** | 10686 | 3573 | 33.4% |
| **≥15** | 9651 | 3235 | 33.5% |

**Supplementary Table 2** The change of long-term survivals according to lymph node count.

| **Total lymph node count** | **Median survival (Months)** | **3-year CCS (%)** | **5-year CCS (%)** | **8-year CCS (%)** |
| --- | --- | --- | --- | --- |
| **≥6** | 54 | 60.4 | 45.9 | 28.1 |
| **≥7** | 54 | 60.7 | 46.2 | 28.3 |
| **≥8** | 55 | 61.1 | 46.4 | 28.6 |
| **≥9** | 55 | 61.6 | 46.7 | 29.4 |
| **≥10** | 56 | 61.8 | 47.0 | 29.5 |
| **≥11** | 56 | 62.1 | 47.3 | 29.8 |
| **≥12** | 57 | 62.6 | 47.8 | 29.9 |
| **≥13** | 58 | 63.0 | 47.9 | 30.1 |
| **≥14** | 58 | 63.2 | 48.1 | 30.5 |
| **≥15** | 59 | 63.9 | 48.9 | 31.0 |


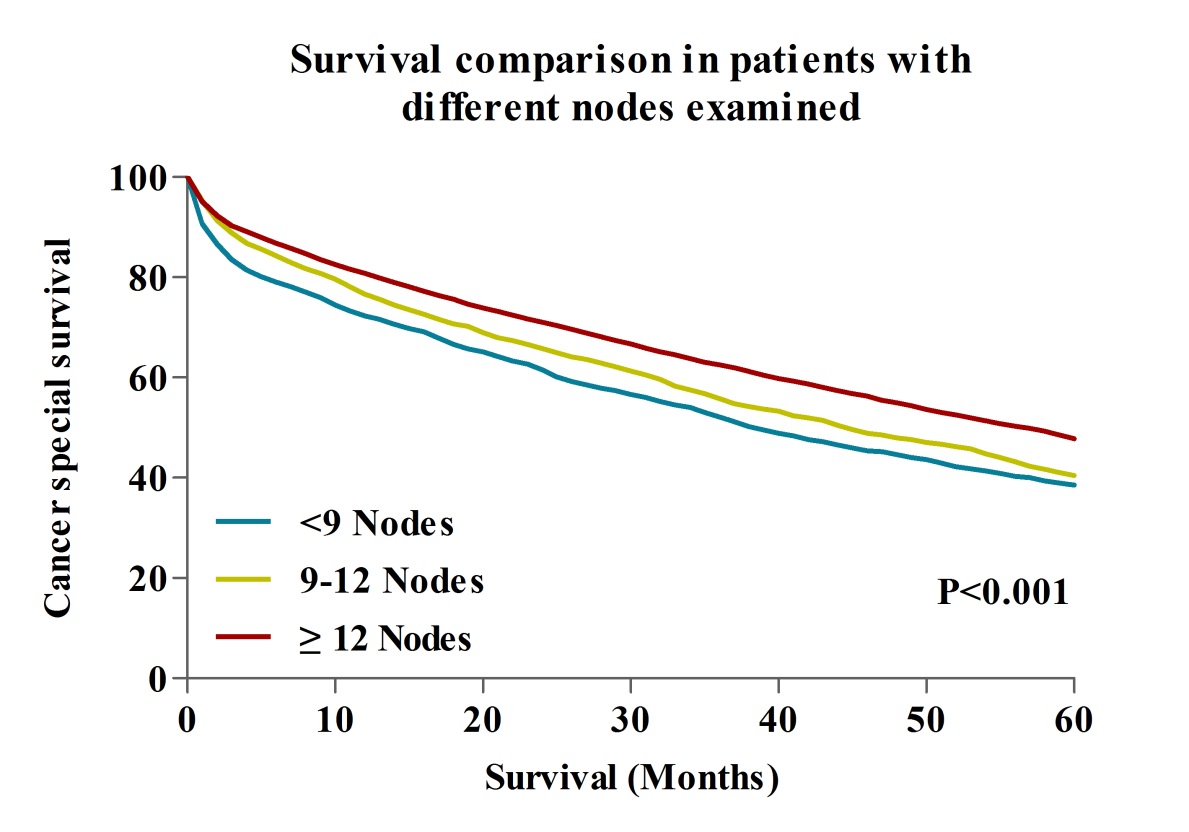


**Supplementary Figure 1**. Survival comparison in patients with different nodes examined.
